# Leachates from plants recently infected by root-feeding nematodes cause increased biomass allocation to roots in neighbouring plants

**DOI:** 10.1038/s41598-021-82022-9

**Published:** 2021-01-27

**Authors:** Peihua Zhang, Dries Bonte, Gerlinde B. De Deyn, Martijn L. Vandegehuchte

**Affiliations:** 1 Terrestrial Ecology Unit, Department of Biology, Ghent University, Karel Lodewijk Ledeganckstraat 35, 9000 Ghent, Belgium; 2 Department of Environmental Sciences, Soil Biology, Wageningen University and Research, Droevendaalsesteeg 3, 6708 PB Wageningen, The Netherlands

**Keywords:** Ecology, Plant sciences

## Abstract

Plants can adjust defence strategies in response to signals from neighbouring plants attacked by aboveground herbivores. Whether similar responses exist to belowground herbivory remains less studied, particularly regarding the spatiotemporal dynamics of such belowground signalling. We grew the grass *Agrostis stolonifera* with or without root-feeding nematodes (*Meloidogyne minor*). Leachates were extracted at different distances from these plants and at different times after inoculation. The leachates were applied to receiver *A. stolonifera* plants, of which root, shoot, and total biomass, root/shoot ratio, shoot height, shoot branch number, maximum rooting depth and root number were measured 3 weeks after leachate application. Receiver plants allocated significantly more biomass to roots when treated with leachates from nematode-inoculated plants at early infection stages. However, receiver plants’ root/shoot ratio was similar when receiving leachates collected at later stages from nematode-infected or control plants. Overall, early-collected leachates reduced growth of receiver plants significantly. Plants recently infected by root-feeding nematodes can thus induce increased root proliferation of neighbouring plants through root-derived compounds. Possible explanations for this response include a better tolerance of anticipated root damage by nematodes or the ability to grow roots away from the nematode-infected soil. Further investigations are still needed to identify the exact mechanisms.

## Introduction

Communication between plants was first observed and reported more than 30 years ago and the number of reported cases has grown rapidly in the recent past^1,2^. There are extensive studies on aboveground chemically mediated plant–plant signaling in the context of herbivore-induced volatiles^3,4^. Volatile organic compounds (VOCs) released from attacked plants can be perceived by neighbour plants of various species. In response to the volatile blends released by emitters, receivers can start expressing genes and synthesizing secondary metabolites involved in plant defences^5^ or can prime their defences against pests^6,7^ suggesting that VOCs play key roles in mediating within- and between-plant signaling above ground^1^.

Chemical signaling among plant roots (including the roots of neighbouring plants) and other soil organisms is often based on root-derived compounds^8^. Root exudates can induce an adaptive interaction between conspecific plants under aboveground herbivore attack by signaling to the herbivore’s enemies, and hence attracting them^9,10^. Root exudates can also carry specific information about the environmental conditions (water stress), growth (flowering timing) and genetic identity (kin recognition) of the donor plants^11–13^. Accordingly, responses such as altered resistance to aboveground herbivory, stomatal aperture and flowering timing are triggered in the receiving/neighbouring plants. However, the effects of root herbivory on plant–plant interactions mediated by root exudates have been less studied than the role of root VOCs in conferring resistance against aboveground herbivores.

Root exudates comprise a compositionally diverse array of different low-molecule secondary metabolites. These metabolites have a multitude of functions in ecological interactions with the soil organism communities^14^. Antimicrobial, insecticide and nematicide compounds in the root exudates act as repellents to pathogens and invaders. Some compounds, such as flavonoids and strigolactones, act as specific signaling molecules mediating the interactions between plants and symbionts in legume-rhizobia and plant-AMF interactions^15,16^. Root exudates can also affect their neighbouring plants negatively by autoinhibition and production of phytotoxins, and positively by kin recognition and resistance to or defence against aboveground herbivores^11,17,18^. Plant growth and biomass allocation to different organs depend on species, ontogeny and on the abiotic (e.g. resource availability) and biotic (e.g. herbivory and competition) conditions experienced by plants^19^. We can therefore anticipate root exudates to carry signals affecting the size and root/shoot ratio of receiver plants. The quantity and quality of root exudates depend on the plant species, the age of individual plants and external biotic and abiotic factors^20,21^. Root exudates can influence their associated root microbiome by mediating symbiotic associations with beneficial microbes or acting in plant defence against pathogenic microorganisms^22^. Furthermore, rhizosphere microbiomes can produce many metabolites which can be taken up by the plants and thus also have an influence on the composition of root exudates^8^. Plants release root exudates that can mediate plant–plant interactions below ground and roots can detect the chemical signals originating from their neighbours. The profile of root exudates can be affected by herbivore attack, yet we currently do not know whether root exudates from a plant attacked by root-feeding organisms can act as a warning signal for neighbouring plants.

Root-knot nematodes (*Meloidgyne* species) are a major group of parasitic nematodes causing the majority of crop damage by parasitic organisms^23^. Root-knot nematodes are obligate parasites that must enter into the roots to feed and reproduce^24^. Juveniles locate plant roots and penetrate them from the root tip, migrate into the vascular cylinder and become swollen and sedentary adults when a permanent feeding site is established. Root-knot nematodes have been known to reduce infected plants’ growth by formation of galls and giant cells in the roots, which can result in a deformed root system^25^. Such a retarded root system severely diminishes plant performance as a reduction in numbers of root branches and root hairs decreases the efficiency of water and nutrient uptake, and thereby imposes water stress and nutrient deficiency^26^.

Aboveground plant–plant signaling mediated by VOCs is shown to have an active distance of 60 cm to 1 m in the field for sagebrush, lima bean and alder^27–29^. Root exudates can change drastically in time and space due to plant development stages and the heterogeneous and complex interactions between plants and soil organisms in the root-soil interface^30^. However, we have little information on belowground root-derived signals in terms of the distance at which plants can receive these signals and actively respond to them. Falik et al*.* (2012) showed that unstressed receiver plants can perceive and propagate the drought stress cues from its drought-stressed neighbour via root exudates^12^. In their study unstressed receiver plants within a distance of 15 cm responded to the root-induced drought stress cues by closing their stomata to increase their readiness to potential future drought stress. Studies of temporal dynamics of root exudates mostly used young plants (up to 4 weeks) in sterilized growing solutions^31^ and therefore lack ecological relevance because of the artificial growing conditions and the absence of interactions with other organisms. Using a split-root system with the creeping bentgrass (*Agrostis stolonifera*) and *Meloidogyne minor* nematodes, we have previously demonstrated that leachates originating from roots varying in their nematode infection status affect the receiver plants differently^32^. Leachates extracted from not-yet infected roots of the nematode-infected plant stimulated the growth of receiver plants significantly compared with leachates from nematode-infected roots of the same donor plant. However, such localized effects may not occur in the field, where the entire mixture of root exudates may reach receiver plants at various distances and different points in time. Thus, the objective of this study is to build upon our previous work and test the role of spatial distance and time since root infection in the effect of the signal-emitting plants on non-infected plants via their root and soil leachates.

We hypothesized that (1) root leachates from nematode-infected plant can act as warning cues for receiver plants; (2) these leachates cause a smaller root system of the receiving plants, similar to the response of nematode-infected plants, as they allocate resources away from attacked organs to prepare for imminent infection by nematodes; (3) this response to the leachates from infected roots will vary in time and space with leachates from locations closer to the infected plant and collected at later times after the infection causing stronger responses of the receiving plants.

## Material and methods

### Study system and experimental design

We used creeping bentgrass, *Agrostis stolonifera*, and the root-feeding nematode species *Meloidogyne minor* as our model system. *M. minor* is known to cause so-called yellow patch disease in creeping bentgrass^33–35^. Creeping bentgrass seeds were obtained from a commercial supplier (Cruydt-Hoeck, Netherlands). Seeds were first surface sterilized: they were treated with 3% household bleach for 10 min, rinsed ten times with distilled water, treated with 10% ethanol for another 10 min and rinsed another ten times with distilled water^36^. The surface-sterilized seeds were placed on wet filter papers in a Petri dish (50 seeds/dish) and left to germinate at 22 °C in a plant breeding room with a light/dark regime of 16 h/8 h. Two-week-old seedlings with an average shoot length of 1.5 cm and average root length of 4 cm were transplanted into the center of the rectangular pots of 70 × 20 × 15.6 cm (length × width × height). The pots were sterilized beforehand by cleaning the surface three times with 95% ethanol and filled with soil. Pots were labelled from number 1 to 30, of which 15 of them were randomly selected after soil fill and plant transplantation for nematode inoculation as treatment pots (T) and water addition as control pots (C). Three pots were then randomly picked out from both groups every 21 days to sample soil for leachates collection.

Most pathogenic nematodes perform well in coarse and oligotrophic soils in Atlantic sandy dunes^37^ and most of the plant-parasitic nematodes that damage turfgrass favor sandy soil^38^. Additionally, plant tolerance to nematode damage decreases as sand content in soil increases because of low water holding capacity and high rate of nutrient leaching^38^. In order to create a soil environment good for both *M. minor* and *A. stolonifera* in this study, we used a mixture of garden soil (Structural, Belgium) and sand (Decor Son, Netherlands) with a volume ratio of 2:1 (garden soil: sand). This soil mixture was sterilized by autoclaving at 121 °C for 30 min before use. According to the product description, the garden soil had 25% of organic matter and a pH of 5–6.5. Electrical conductivity was 300 μs/cm. It also had 1.25 kg/m^3^ composite NPK (14-16-18) fertilizer mixed in the garden soil.

Plants were inoculated with nematodes 1 week after transplanting. Twenty ml of nutrient supplement (2 g of the product in 1 L distilled water, COMPO, NPK:16-9-20) was added to the central plants in both treatment and control group before the nematode inoculation. The root-knot nematodes (second-stage juveniles J2) cultured on potatoes were purchased from HZPC, Netherlands. Four ml of the inoculum of nematodes in tap water with a concentration of about 350 J2 individuals/ml was added into a hole at 0.5 cm distance from the stem and 2 cm deep below the soil surface of the treatment plants to standardize the inoculation procedure and maximize the infection rate. There were approximately 1400 juveniles per treatment plant, which is more than the maximum density (374 J2 per 100 cm^3^ soil) of J2 found in roots of infected *A. stolonifera* in the field^35^. We used a higher density to compensate for anticipated losses of nematodes after inoculation because of establishment failure. The same amount of water (4 ml) was added into the rhizosphere of the control plants. The pots were organized in a random configuration with a light/dark regime of 16 h/8 h and 22 °C constant room temperature. During the experiment the water content of the soil mixture was maintained at volume-based 31–32% by weighing the pots every 3 days and resetting to the initial weight of the experimental unit by adding water equally into the seven compartments.

**Figure 1:**
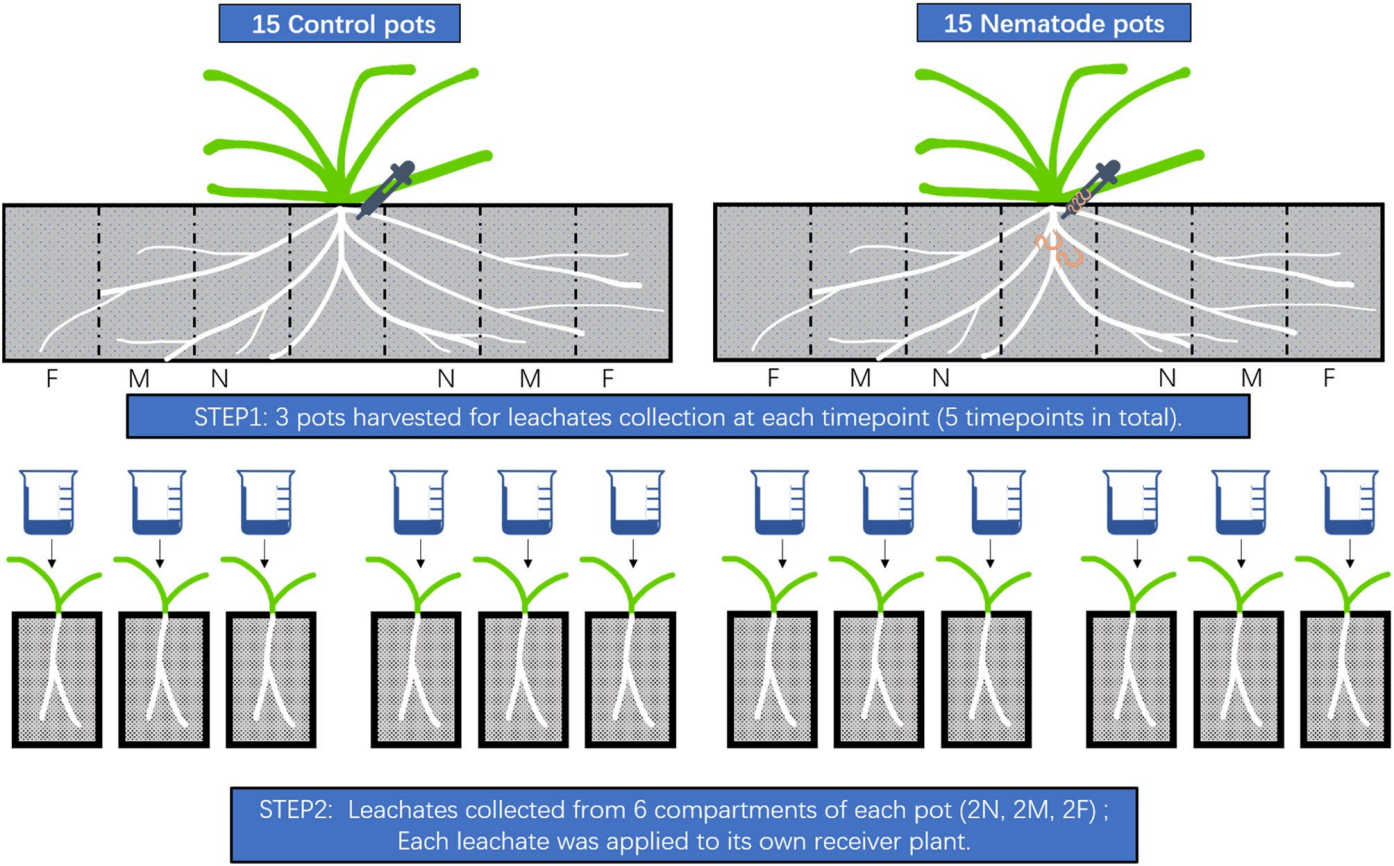
Schematic diagram of the experiment: Step 1 indicates three pots were randomly selected from the control and nematode-treated group at each timepoint to collect leachates from harvested samples of root and soil at 5–15 cm (N),15–25 cm (M) and 25–35 cm (F) distance away from the central donor plants (nematode inoculum addition as treatment and water addition as control); Step 2 indicates that leachates were collected from the harvested sample and were then applied to receiver plants. Each distance had 6 receiver plants as replicates at each sampling timepoint for each treatment group (3 pots* 2 sides). The response variables measured in the receiver plants were root, shoot, and total biomass, root/shoot ratio, shoot height, shoot tiller number, rooting depth and root number.

In each rectangular pot, seven compartments with an equal length of 10 cm were delineated lengthwise for future soil and root sampling (See Fig. 1). As the plant was present in the central compartment, the three compartments on each side of the central one represent three distance classes from the central plant (near, middle and far). Compartments were only marked on the pot, so that soils were not physically separated between adjacent compartments. Three pots from each group (control and nematode addition treatment) were randomly selected for root and soil collection on day 21, 42, 63, 84 and 105 after the nematode inoculation. As we have little information on how *M. minor* reproduces in *A. stolonifera* plants, these collection time points are based on the well-known life cycle of *M. minor* on potatoes where females with egg masses typically occur 6 weeks after inoculation with J2 juveniles^34^. The soil containing roots was harvested from all compartments except for the central compartment where the seedling was planted. The collected soil was stored at 4 °C before use for a maximum of 1 week. The collected root and soil samples were used to extract leachates to treat the receiver plants. In total, there were 180 replicates (2 treatments × 5 time points × 3 pots × 3 distances × 2 sides). We consider the replication for the interaction between all factors sufficient given that the aim of this experiment was proof of concept. After each root and soil collection, new soil was added and new neighbouring plants planted on both sides of each central plant for a follow-up experiment, which lasted for another 30 days. At the end of these additional 30 days, during which nematodes were left to reproduce in the pots, we collected 15 g of rhizosphere soil and 20 g of bulk soil, from which we extracted nematodes using Baermann funnels^39^ and counted them. Nematode abundance in the soil samples on day 51, 72, 93, 114 and 135 after the nematode inoculation is listed in supplementary material Table 1. The identification of the nematodes was based on morphological differences in their bulbs, feeding apparatus and locomotion. The consistent appearance of nematodes in the soil proved that the inoculation was successful. The detection of new mobile juveniles in the soil even 135 days after the inoculation date suggested successful reproduction and possible new infections of the donor plant.

**Table 1:** Measured traits and lambda value used to transform the data.

Phenotypic traits	Lambda value
Total biomass	0.525
Root/shoot ratio	− 0.7
Shoot biomass	0.525
Shoot height	**Not transformed**
Number of shoot tillers	0.5
Maximum rooting depth	1.45
Number of roots	**Not transformed**

P < 0.05 is highlighted in bold.

### Preparation of leachates

Leachates from the root and soil samples harvested from the donor plants were collected three times in total (once a week during the 3 weeks of receiver plant growth). At each collection time, 400 g of the harvested root and soil samples were weighed into pots with perforations in the bottom, which were firstly saturated with distilled water, after which approximately 240 ml of distilled water was poured into each pot and 120 ml of leachates was collected^40^. The leachates were then filtered through filter paper with a pore size of 20 μm (Whatman, Quantitative filter papers, ashless grades, grade 41) before use to remove nematodes potentially present in the leachates. Filtrated leachates were checked for nematodes under the microscope during the experiment by randomly selecting 6 samples out of the 18 samples at the first and third time point. In addition, 15 leachate samples were checked in another experiment using the same filtration procedure^32^. No nematodes were detected in any of the checked samples. Receiver plants were then treated with the filtrated leachates for 3 weeks. In the first week, 40 ml of leachates were applied carefully with a pipette at a distance of 0.5 cm from the stem of the receiver plants three times and two times 60 ml per week in the latter 2 weeks. This change in applied leachate volume was to avoid damage caused by adding large quantities of leachates to the small seedlings at the start of the experiment. During the experiment the stocks of root and soil leachates were stored in sealed plastic bottles at 4 °C for maximum 1 week.

### Receiver plants

The receiver plants were *A. stolonifera* from the same batch of seeds, which were surface sterilized as described above. The seeds of these receiver plants were germinated 2 weeks before each root and soil collection time of the leachate donor plants. The receiver plants were put into cylindrical pots (13.5 cm in height with a diameter of 4.5 cm) with 1 L of the same mixed and sterilized soil. The receiver plants were placed in random order in another plant breeding room with a light/dark regime of 16 h/8 h and 22 °C constant room temperature to exclude the potential effect of VOCs from the nematode-infected plants. Receiver plants were harvested after 3 weeks of growth since transplantation. Root, shoot, and total biomass, root/shoot ratio, shoot height, shoot tiller number, maximum rooting depth and root number were quantified. Fresh roots were cleaned and scanned using an EPSON scanner (Epson Expression 11000 XL). Image J was used to count the first-order roots and measure the maximum root depth by analyzing the scans of the roots. After the scanning, root samples were dried at 70 °C for 72 h before weighing. Fresh shoots were cleaned before measuring the length and tiller numbers. Shoots were dried at 70 °C for 72 h before weighing.

### Statistical analysis

The effects of leachate origin, i.e. from plants inoculated with nematodes or nematode-free plants, sample collection distance from the leachate donor plant and number of days since the nematode inoculation on receiver plants’ root, shoot, and total biomass, root/shoot mass ratio, shoot height, shoot tiller number, rooting depth and root number were analyzed with mixed effect models, using the lme4 package in R. Response variables were transformed to meet assumptions if needed (See Table 1). All predictors were analyzed as categorical variables. Time of leachates collection has 5 levels as 21, 42, 63, 84, 105 days after the nematode inoculation. Distance from the donor plants of samples collected for leachate extraction has 3 levels as 5–15, 15–25, 25–35 cm away from the central plant. Treatment has 2 levels: nematode inoculation treatment (T) and control (C). Treatment, Time and Distance, as well as their two-way interactions and the three-way interaction were included as fixed effects in our model. Pot was included as random effect. Shapiro–Wilk normality tests were used to check for normal distribution of the residuals. Residuals were plotted after analyses and found to be (approximately) normally distributed and homogeneous, except for root biomass, total biomass, root/shoot ratio, shoot tiller number and maximum rooting depth. The latter four variables were transformed using Tukey's Ladder of Powers to produce more normally distributed residuals (transformTukey function, rcompanion package). The function simply loops through lambda values and then chooses the lambda that maximizes the Shapiro–Wilks W statistic. The lambda values are listed in Table 1. Only transformed root/shoot ratio was found to not satisfy the normal distribution with a *p* value of 0.03486 for the Shapiro–Wilk normality test. Given the overconservative nature of this test, this small deviation from normality should not affect interpretation of results. Root biomass was analysed using identical linear models, except that *p* values were calculated based on parametric bootstrapping (R package afex) since even the transformed root biomass residuals still did not satisfy the normality assumption (Shapiro–Wilk normality test, *p* value = 0.0054).

## Results

### Spatiotemporal change in leachate effect on root/shoot ratio of the receiver plants

**Figure 2:**
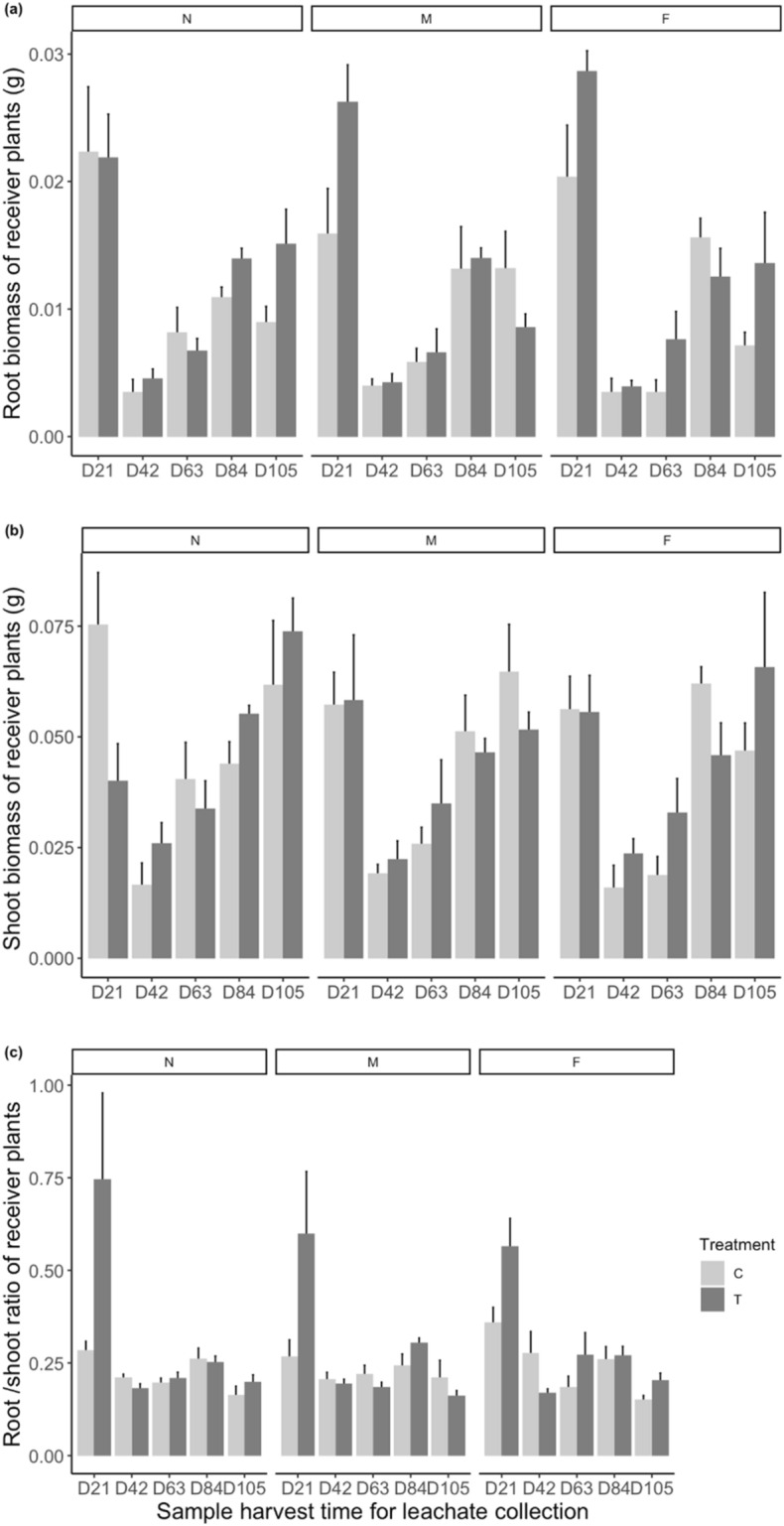
Effect of timing of leachate collection, location of leachate collection and nematode infection on the root biomass (**a**), shoot biomass (**b**) and root/shoot ratio (**c**) of receiver plants. T = leachates from nematode-infected plants, C = leachates from control plants; D21, D42, D63, D84, D105 indicate the leachate collection time in number of days (D) after nematode inoculation or water addition. N, M and F indicate the distances from the focal plant for leachate collection (N = 5–15 cm, M = 15–25 cm, F = 25–35 cm). Bars are means ± SE (n = 6).

We found that both nematode treatment of the leachate donor plant (F_1,19.99_ = 5.23 *p* = 0.03) and time of soil sampling for leachate collection (F_4,19.989_ = 23.0474; *p* < 0.001) had significant and interactive effects (F_4,19.989_ = 3.78 *p* = 0.019) on the root/shoot ratio of leachate-receiving plants (See Table 2). Meanwhile, distance from the leachate donor plant had interactive effects with nematode treatment and time of sample collection on receiver plants’ root/shoot ratio (F_8,129.153_ = 2.20 *p* = 0.03). The root/shoot ratio of plants that received leachates from nematode-infected plants was significantly higher than that of plants receiving leachates from control plants, but only when those leachates were collected at the first sampling time. The root/shoot ratio change over time showed a different pattern between plants receiving leachates from nematode-infected plants and from control plants (Fig. 2c). Plants treated with leachates from nematode-infected donors had a significant decrease in root/shoot ratio from the second leachate collection time onwards while plants receiving leachates from control donors only had a significant decrease at the third and last time point (Fig. 2c). Patterns of root/shoot ratio were similar for plants receiving leachates collected at each of the three distances from the donor plant. However, the difference between plants receiving leachates collected at the first sampling time from nematode-infected or control plants was largest when leachates were collected closer to the donor plant. This significant decrease in root/shoot ratio in plants treated with early-collected leachates from nematode-inoculated plants was the result of the significantly larger root biomass (Table 3) and relatively smaller shoot biomass (although not significantly different) in receiver plants treated with leachates from nematode-infected donors than in plants treated with leachates from control donors (Fig. 2a, b).

**Table 2:** Effect of nematode inoculation, distance from the focal plant, and sample harvest time for leachate collection on measured traits of receiver plants.

Factor	Num Df	Den Df	F	*p*
**Root/shoot ratio**				
Nematodes	1	19.992	5.2287	**0.033**
Time	4	19.989	23.0474	**3.00e**−**07**
Distance	2	129.159	0.3559	0.70
Nematodes × time	4	19.989	3.7783	0.019
Nematodes × distance	2	129.159	0.1170	0.88
Time × distance	8	129.153	0.3619	0.93
Nematodes × time × distance	8	129.153	2.1986	0.031
**Total biomass**				
Nematodes	1	19.993	0.8826	0.35
Time	4	19.991	28.5500	**5.16e**−**08**
Distance	2	129.150	0.8271	0.43
Nematodes × time	4	19.991	0.5929	0.67
Nematodes × distance	2	129.150	0.5591	0.57
Time × distance	8	129.143	0.5763	0.79
Nematodes × time × distance	8	129.143	1.8892	0.066
**Shoot biomass**				
Nematodes	1	19.993	0.1733	0.68
Time	4	19.991	21.0037	**6.29e**−**07**
Distance	2	129.150	0.9911	0.37
Nematodes × time	4	19.991	1.1851	0.34
Nematodes × distance	2	129.150	0.5349	0.58
Time × distance	8	129.144	0.5510	0.81
Nematodes × time × distance	8	129.144	1.7488	0.093
**Shoot height**				
Nematodes	1	19.980	0.8826	0.31
Time	4	19.974	28.5500	**5.65e**−**05**
Distance	2	129.244	0.8271	0.69
Nematodes × time	4	19.974	0.5929	0.088
Nematodes × distance	2	129.244	0.5591	0.54
Time × distance	8	129.234	0.5763	0.59
Nematodes × time × distance	8	129.234	1.8892	0.65
**Maximum rooting depth**				
Nematodes	1	19.985	0.8826	0.25
Time	4	19.981	28.5500	**2.85e**−**06**
Distance	2	129.210	0.8271	0.99
Nematodes × time	4	19.981	0.5929	0.33
Nematodes × Distance	2	129.210	0.5591	0.76
Time × distance	8	129.202	0.5763	0.23
Nematodes × time × distance	8	129.202	1.8892	0.20
**Shoot tiller number**				
Nematodes	1	19.991	0.8826	0.77
Time	4	19.988	28.5500	**2.47e**−**05**
Distance	2	129.169	0.8271	0.48
Nematodes × time	4	19.988	0.5929	0.53
Nematodes × distance	2	129.169	0.5591	***0.053***
Time × distance	8	129.162	0.5763	0.48
Nematodes × time × distance	8	129.162	1.8892	0.87
**Root number**				
Nematodes	1	19.980	0.8826	0.30
Time	4	19.975	28.5500	**9.12e**−**07**
Distance	2	129.241	0.8271	0.69
Nematodes × time	4	19.975	0.5929	***0.057***
Nematodes × distance	2	129.241	0.5591	0.43
Time × distance	8	129.232	0.5763	0.49
Nematodes × time × distance	8	129.232	1.8892	0.62

P < 0.05 is highlighted in bold. P ≥ 0.05 is highlighted in bolditalics.

**Table 3:** Effect of nematode inoculation, distance from the focal plant, and sample harvest time for leachate collection on receivers’ root biomass.

Factor	Pr (> PB)
**Root biomass**	
Nematodes	**0.011**
Time	**0.00049**
Distance	0.89
Nematodes × time	0.17
Nematodes × distance	0.62
Time × distance	0.85
Nematodes × time × distance	**0.038**

*p* values were calculated based on parametric bootstrapping.

P < 0.05 is highlighted in bold.

### Temporal change in leachate effects on receiver plants’ total biomass

**Figure 3:**
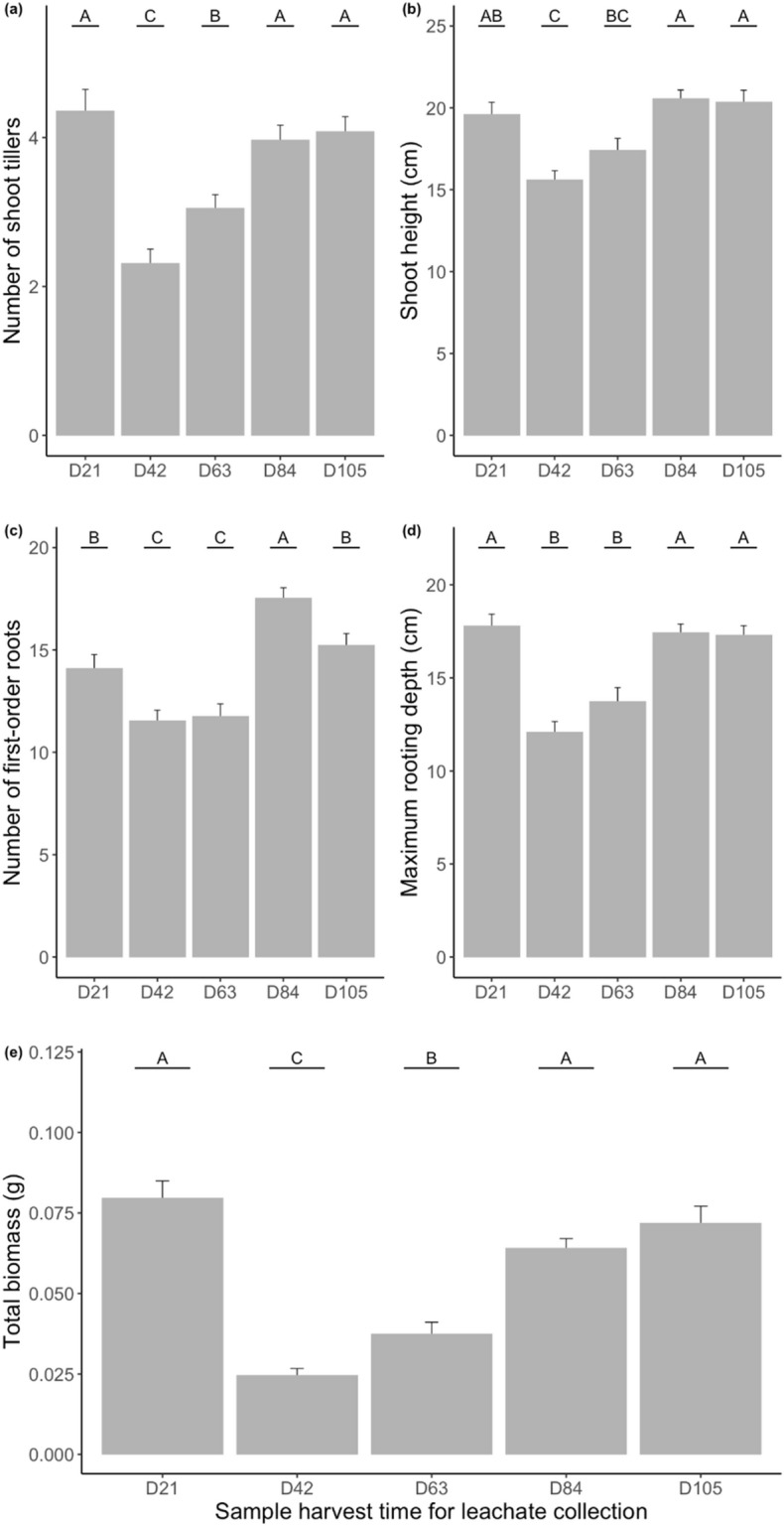
Effect of timing of leachate collection on the number of shoot tillers (**a**), shoot height (**b**), number of first-order roots (**c**), maximum rooting depth (**d**) and total biomass of the entire plant (**e**) in plants receiving leachates from donor plants with or without root-knot nematodes (bars represent averages across nematode and control treatments and across sampling distances). D21, D42, D63, D84, D105 indicate the leachate collection time in number of days (D) after nematode inoculation or water addition. Different letters above the means indicate significant differences between treatments based on Tukey multiple comparison test. Bars are mean ± SE (n = 36).

We found that only the time of soil sampling for leachate collection from the donor plants (F_4,19.991_ = 28.55; *p* < 0.001) had a significant effect on the total biomass of receiver plants (Table 2). The general trend for total biomass, shown for all distances and both the control and treatment group (Fig. 3e), was to be strongly inhibited by 70% by leachates from the second and inhibited by 50% from the third collection time (42 and 63 days) compared with receiver plants treated with leachates from the first collection time (21 days). Plants treated with leachates collected at the two latest time points (84 and 105 days) had a similar total biomass as plants that received leachates from the first time point.

### Temporal change in leachate effect on aboveground plant size

We found that the time of soil sampling for leachate collection (F_4,19.974_ = 28.55; F_4,19.988_ = 28.55; *p* < 0.001) had a significant effect on shoot height and shoot tiller number (Table 2). Leachates collected at the second and third time point (42 and 63 days) had a significant inhibitory effect on shoot tiller number of the receiver plants (Fig. 3a). Leachates collected at the second time point (42 days) had a significant inhibitory effect on shoot height of the receiver plants (Fig. 3b).

**Figure 4:**
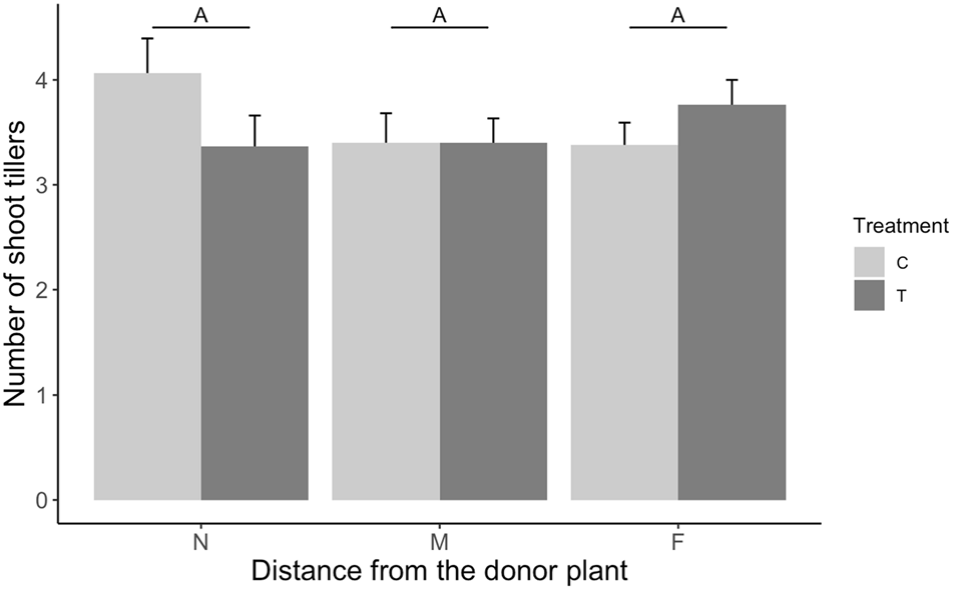
Effect of location of leachate collection on number of shoot tillers of receiver plants. T = leachates from nematode-infected plants, C = leachates from control plants. N (5–15 cm), M (15–25 cm), F (25–35 cm) indicates different distances away from the leachate donor plants. Bars are means ± SE (n = 30) across all leachate collection times. The same letter above the means indicate no significant differences between distances based on Tukey multiple comparison test.

A close-to-significant interactive effect (F_2,129.169_ = 0.5591; *p* = 0.053) of nematode inoculation of the donor plants and distance of the leachate collection from the donor plants was found on shoot tiller number of the receiver plants (Fig. 4). Receiver plants treated with leachates from nematode-infected donors (T) had fewer shoot tillers when receiving leachates collected at the closest distance from the donor plants than plants treated with leachates from control plants (C). However, shoot tiller number of receiver plants treated with leachates from nematode-infected plants increased when leachates were collected at the furthest distance from the donor plants. At this distance, their shoot tiller number was higher than that of receiver plants receiving control leachates.

### Temporal change in leachate effect on belowground root development

Time of soil sampling for leachate collection from the donor plants (F_4,19.981_ = 28.55; F_4,19.975_ = 28.55; *p* < 0.001) had a significant effect on maximum rooting depth and root number (Table 2). The general trend, shown for all distances and both the control and treatment group (Fig. 3c, d), was for root number and maximum rooting depth to be strongly inhibited by leachates from the second and third collection time (42 & 63 days). Then, inhibition was gradually lost from the fourth time (84 days) onwards and the maximum rooting depth of plants treated with leachates collected at the latest two time points (84 and 105 days) reached the same level as those treated with leachates from the first time point.

**Figure 5:**
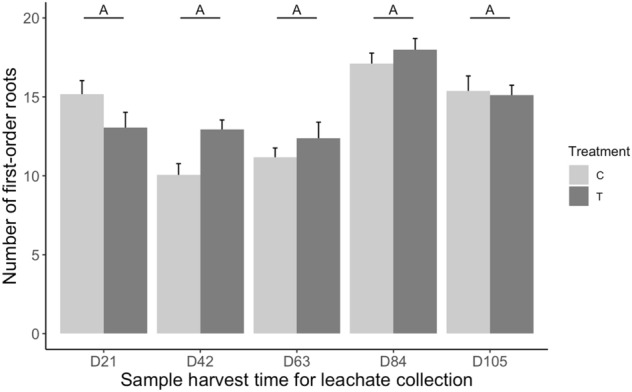
Effect of timing of leachate collection on number of first-order roots of receiver plants. T = leachates from nematode-infected plants, C = leachates from control plants. D21, D42, D63, D84, D105 indicate the leachate collection time in number of days (D) after nematode inoculation or water addition. Bars are means ± SE (n = 18) across all leachate collection locations. The same letter above the means indicate no significant differences between leachate collection times based on Tukey multiple comparison test.

There was a trend for the temporal change in root number to differ between plants receiving leachates from nematode-infected plants and those receiving control leachates (Nematodes by Time interaction: F_4,19.975_ = 0.59 *p* = 0.057, Fig. 5). Here, the root number of receiver plants treated with leachates from nematode-infected plants stayed relatively similar when treated with leachates collected from the first three time points (21, 42 and 63 days) and increased at the latest two time points (84 and 105 days). Receiver plants treated with control leachates had a decrease in root number when treated with leachates collected from the second and third time points (42 and 63 days) compared with leachates form the first time point and then increased to the same level of receiver plants treated with leachates from nematode-infected plants at the latest two time points (84 and 105 days).

## Discussion

In this study, we set out to investigate whether nematode-infected plants can use root-derived compounds to warn neighbouring non-infected plants to change their phenotype in preparation for future nematode infection. We also aimed to test how certain responses to the root-derived compounds vary with the distance from the compound-releasing plant and with time since nematode infection. We found that receiver plants responded to early-collected leachates from nematode-infected donor plants by allocating more biomass to their roots. This is a different response compared to that of plants exposed to direct root herbivory, which allocate less resources to the attacked organs, i.e., their roots^40–44^. Contrary to our hypothesis, plants exposed to leachates from infected plants were more influenced by leachates collected at early times than at later times. Receiving plants generally showed similar responses to leachates collected from sites both close and far from the central donor plant, regardless of nematode inoculation.

Plants exposed to leachates from nematode-infected plants were not significantly different from plants exposed to leachates from control plants in most of the measured traits except for root/shoot ratio. Altering biomass and resource allocation pattern is one of the mechanisms of plants’ compensatory adjustment to cope with herbivory and has been documented in many plant species (including crops and trees), all of which show an increased transportation of photo assimilates or N into roots and stems from the leaves upon leaf herbivory^41–44^. A similar pattern was found in some studies belowground regarding direct root herbivory with the diversion of resources away from the attacked tissues to organs that are inaccessible to foraging herbivores. Newingham, Callaway and Bassirirad (2007) found that *Centaurea maculosa* infested with *Agapeta zoegana* (root-boring herbivore) maintained shoot N status by shifting more of the acquired N from the root to the shoot^45^. Another study using radioactive ^11^CO_2_ showed that root-attacked maize plants allocate more newly assimilated ^11^C carbon from source leaves to stems instead of roots^46^. Contrary to the patterns of resource reallocation in plants with direct root herbivory, plants with no direct contact with root-knot nematodes that receive root-derived signals (leachates) from plants of which the roots were attacked seem to prepare for potential root attack by temporarily investing more in their root growth, as we also observed in our previous split-root experiment^32^. Perhaps the relatively larger biomass of the root system is a form of insurance, compensating for loss of functionality in attacked roots.

Root/shoot ratio was the only trait that responded differently to leachates from nematode-inoculated plants than to leachates from nematode-free control plants. Plants receiving leachates from nematode-infected plants collected at a short time of 21 days after inoculation showed a significant diversion of resources from shoot to root compared with receiver plants treated with leachates from nematode-free control plants when there was no damage done to the central plant roots. At our first sampling time, central plants had a small root system, which had not yet extended into the closest compartments. Leachates collected at the first timepoint mainly contained the root diffusates from the central roots. However, later collected leachates can contain leaked cell contents due to the mechanical damage of the roots of the central plants which had by then proliferated into the other compartments. Leakage of cell contents from the wound sites may have altered the composition of the leachates collected at later times and caused a different response in the leachate-receiving plants. Root-feeding nematodes can change the profile of root exudates derived from the infected roots. Studies have shown that roots infected with *Meloidogyne incognita* act as metabolite sinks, which results in increased leakage into the rhizosphere compared with healthy roots^47–50^. Carbohydrates were found to be the major organic compounds in early exudates while nitrogenous compounds became the major organic compounds leaking from roots after 14 days of nematode infection^47^. The authors suggested that increased amylase, cellulase and pectinase production by the nematodes before the permanent feeding site was established may contribute to the high level of carbohydrates in the early exudates. Nitrogenous waste products and secreted stylet exudates from the adult female nematodes may contribute to the increased nitrogenous compounds in the later root exudates. Analysis of stylet exudates from adult female *Meloidogyne incognita* revealed presence of a mixture of proteins in the stylet exudates^51^. These parasitic nematodes produced and secreted compounds that play key roles in establishment of feeding sites and activation of defensive responses in plants^24,52,53^.

This observation of a higher root/shoot biomass ratio is in line with results from a previous experiment in which the root system of a single *A. stolonifera* plant was split into a nematode-infected and nematode-free half^32^. Leachates from the nematode-free root compartment of the infected plant similarly triggered an increased allocation of biomass to the roots. In this previous experiment, we can exclude compounds originating from the nematodes as a mechanism, as this change in root biomass allocation was not observed in plants receiving leachates from the nematode-infected half of the donor plant’s root system. We did not specifically analyze the compositional differences in the leachates from soil with nematode-free plants and with nematode-infected plants, as this was outside the scope of this first proof of concept of root feeders influencing belowground plant-plant communication. Taking the two studies together, we can speculate that root-derived compounds from nematode-infected plants can act as a warning signal, causing the receiving plants to allocate more resources to their roots. Altering biomass allocation after receiving leachates from root-attacked plants might be a mechanism for plants to cope with future root herbivory. However, the signal caused by root-knot nematodes’ feeding may have been counteracted by the mechanical damage of the roots at later stages in this experiment, thus inducing a different response of root/shoot ratio in receiver plants at these later points in time. Future studies should focus on the chemical composition of soil leachates or root exudates to distinguish the source of the signal (transmitted by nematodes or plant roots) and reveal mechanisms underlying the changes we observed in receiver plants. While autoclaving our soil may have influenced its chemical composition, especially in relation to VOCs, this effect would have been similar for nematode-infected and -free plants as they were both grown in autoclaved soil. Nevertheless, the autoclaving process may have induced chemical processes not typically found in field soils.

Receiver plants had fewer shoot tillers when treated with leachates collected at sites at close distance from the nematode-inoculated donor plants compared with plants treated with leachates collected at the same distance from the nematode-free donor plants. However, shoot tiller number of receiver plants increased when treated with leachates collected at sites at a far distance from the nematode-inoculated donor plants and was relatively higher than that of receiver plants treated with the leachates collected at this far distance from nematode-free donor plants. This might be due to the dilution of signals in the leachates collected at further distances from the control donor plants. Inoculation with nematodes may have caused a different composition of root-derived compounds, which most actively affected leachate-receiving plants at close sites by reduced shoot tiller number.

All measured plant traits were significantly impacted by the timing of the leachate collection, so whether they were collected from young or older plants. The inhibitory effect of early-stage leachates was gradually lost when receiver plants were treated with leachates from later times. Apart from the older plants being more likely to incur root damage by the sampling procedure, as described above, these effects could also be caused by ontogenetic changes in the donor plant as receiver plants were always of the same age. Older plants allocate less C to roots than young plants, which results in a reduced exudation per unit root biomass in older plants^54^. Meharg and Killham (1990) showed that perennial ryegrass grown under field conditions translocated 67% of assimilated ^14^C into belowground tissues after 4 weeks of growing and this percentage dropped to 14% after growing for 24 weeks^55^. The plant-age hypothesis in optimal-defence theory predicts that extrinsic factors such as selection by herbivores lead to high levels of defence in juveniles, followed by decreases as plants mature and become less susceptible to the fitness reductions of these attacks^56^. In our case, early-time leachates were collected from younger plants and might have responded most strongly to nematode infection. Thus, a stronger response can occur in leachate-receiving plants to leachates collected from younger donor plants due to a larger acquisition of resources and their allocation to defensive compounds, which can end up in the exudates of juvenile plants.

In addition to these anticipated ontogenetic changes in exudates, interactions among plant-feeding nematodes, bacteria-feeding nematodes and microbes can also play a role in determining the net effect on the leachate-receiving plants. We used sterilized soil for the experiment but cannot keep soils sterile for such a long period (105 days). Therefore, microbial populations in our soils could have increased over time and affected the root exudates in the soil from which leachates were sampled at later time points. Higher mineralization of root exudates by the increased microbial activity may also counteract the inhibitory effect of plant defensive compounds on the plants receiving leachates collected at later times from more mature plants. Moreover, despite that bacteria-feeding nematodes were rare in our initial inoculum (1 *Cephalobidae* found in 2000 *M. minor* juveniles), their abundance increased over time during the whole experiment (in the Nematode treatment only, Supplementary Table 1). Studies have shown that low-level herbivory by plant-parasitic nematodes (around 1000 individuals per plant) can stimulate the growth of plants due to the interactions among plant-feeding nematodes, bacteria-feeding nematodes and microbes^50,57–59^. This stimulated growth in plants was partly due to the enhanced microbial growth and activity in response to the leakage of rhizodeposits due to the root damage by plant-parasitic nematodes. Bacteria-feeding nematodes, on the other hand, then release the nutrients by grazing on these microbes, which in turn stimulate the growth of plants. We used 1400 juveniles of *M. minor* per treatment plant in this study and it is possible that this enhanced growth also occurred in our plants. Whether plants stop sending the “warning” signal when the constrained growth caused by plant-feeding nematodes is mitigated by the enhanced growth due to the interactions among nematodes and microbes requires further investigation. Furthermore, responses in control receiver plants and treatment receiver plants did no longer change after the first timepoints even though the abundance of bacteria-feeding nematodes kept increasing over time. This strongly suggests that the transient differences observed between control and treatment receiver plants were not due to the bacteria-feeding nematodes. We argue that independent of the mechanisms, the interactions we observed are ecologically more relevant compared to results of studies using young plants of less than 4 weeks in sterilized growing solutions over a relatively short time^31^.

The plant response induced by herbivory, mechanical damage, pathogens and nematodes can be different due to, amongst others, differences in the severity and quantity of the damage. However, the perception of signals emitted by plants experiencing each of these types of root damage can trigger the same stereotypical defence response^60^. Damage-induced plant defence response and herbivory-induced plant defence response have been discussed and compared in detail regarding aboveground VOCs^61,62^. Mithöfer et al. (2005) found that mechanical damage resembling herbivory was sufficient to elicit similar plant responses, such as herbivory-related volatile emission after herbivore feeding^63^. In a molecular analysis of poplar defence against herbivory, severe mechanical wounding with pliers could elicit even higher amounts of expression and larger numbers of significantly induced genes^64^. In our sampling process, we needed to sample the soil that contained roots at different distances from the central donor plant by cutting, thus causing mechanical damage to the root system. The similar effect of leachates from nematode-infected plants and control plants could suggest that a similar response can be induced in roots by mechanical damage or nematode damage for many plant traits. However, our observed effects of leachates from nematode-infected plants on the root/shoot ratio of receiver plants highlights that this is not the case for all plant traits.

We used root-soil leachates containing root exudates and leachate-receiving plants representing neighbour plants to study whether root exudates from a nematode-infected plant can act as a signal to warn neighbouring plants. Our results show that root/shoot ratio is the main responsive trait and we can interpret the response as a preparation for future nematode attack. In contrast to documented increases in allocation to aboveground tissues after plant’s roots are directly attacked, we here show that such plants with attacked roots send a signal causing their neighbors to temporarily increase their allocation of biomass to roots. We speculate that this changed allocation may present an informed escape strategy to avoid the nematode attack in the short term by allocating more resources to roots to grow away from the infection zone, enabled by a larger root biomass divided over a smaller number of roots. Nevertheless, more investigations are needed to compare the effect size of the sole effect of the plant feeders, interactions among nematodes and microbes and mechanical damage to unravel the mechanisms underlying the results of this study.

## Supplementary Information

Supplementary Table 1.
