# Supplementary material for: Leachates from plants recently infected by root-feeding nematodes cause increased biomass allocation to roots in neighbouring plants
Source: Sci Rep. 2021 Jan 27;11:2347. doi: 10.1038/s41598-021-82022-9 (PMC7840926; doi:10.1038/s41598-021-82022-9)
Supplement: Supplementary file 1 — Supplementary Table 1. [file 41598_2021_82022_MOESM1_ESM.pdf]

Leachates from plants recently infected by root-feeding nematodes cause increased biomass allocation to roots in neighbouring plants

Peihua Zhang[1,*], Dries Bonte[1], Gerlinde De Deyn[2], Martijn L. Vandegehuchte (ORCID: 0000-0003-1283-4654)[1]

[1]Ghent University, Department of Biology, Terrestrial Ecology Unit, Karel Lodewijk Ledeganckstraat 35, 9000 Ghent, Belgium

[2]Wageningen University & Research, Department of Environmental Sciences, Soil Biology, Droevendaalsesteeg 3, 6708PB Wageningen, Netherlands

[*]Corresponding author: peihua.zhang@ugent.be

Table: Nematode abundance count from 35 grams of soil

Source plant	Timepoint	Plant feeders	Bacteria feeders	Days after inoculation
T9	1	8	396	51
T12	1	2	339	51
T27	1	9	327	51
T11	2	0	4026	**86**
T17	2	0	3473	**86**
T26	2	0	2520	**86**
T4	3	21	4242	93
T8	3	0	4299	93
T30	3	5	4311	93
T2	4	0	8424	114
T14	4	0	13177	114
T15	4	148	4991	114
T6	5	2	7347	135
T18	5	1	6086	135
T25	5	34	7792	135

Plant feeders refer to *Meloidogyne minor* and bacteria feeders refer to *Cephalobidae* species; the second timepoint for nematode counting was delayed by two weeks (86 days instead of the planned 72 days) due to the bad growth of seedlings needed in our follow-up experiment, which necessitated the growing of a new batch of seedlings and the refilling of the trays only when this new batch was ready for transplantation.
